# Diagnostic Test Accuracy of Artificial Intelligence in Large Vessel Occlusion: A Systematic Review and Meta‐Analysis

**DOI:** 10.1155/nri/5709868

**Published:** 2026-04-21

**Authors:** Lydia Susanti, Kevin N. Cuandra, Christopher Daniel Tristan, Muhammad Zaed Fatahillah, Alifya Rayyani Shofiy, Noel Matthew Imaniku Sihombing, Sandra Rosa Uli Siahaan, Livilia Abigail Onggowasito, I. Komang Tri Yasa Widnyana, Sayidah Alfiah Ziaur Rahmah, Amanda Yulita Amalia, Zahra Roidah Amalia Hasna, Zaki Sidqi Aaliyya, Andi Sitti Nur Fatimah Madaeng, Muhammad Naufal Hibatullah, Nathania Augustine Kristaningtyas

**Affiliations:** ^1^ Department of Neurology, Faculty of Medicine, Andalas University, Padang, Indonesia, unand.ac.id; ^2^ Department of Medicine, Faculty of Medicine, Andalas University, Padang, Indonesia, unand.ac.id; ^3^ Department of Medicine, Faculty of Medicine, Sebelas Maret University, Surakarta, Indonesia, uns.ac.id; ^4^ Department of Medicine, Faculty of Medicine, Islamic University of Indonesia, Yogyakarta, Indonesia, uii.ac.id; ^5^ Department of Medicine, Faculty of Medicine, Veteran National Development University (UPNVJ), Jakarta, Indonesia; ^6^ Department of Medicine, Faculty of Medicine, Sumatra Utara University, Medan, Indonesia; ^7^ Department of Medicine, Faculty of Medicine, Ganesha University of Education, Bali, Indonesia, undiksha.ac.id; ^8^ Department of Medicine, Faculty of Medicine, Jenderal Soedirman University, Purwokerto, Indonesia, unsoed.ac.id; ^9^ Department of Medicine, Faculty of Medicine, Hasanuddin University, Makassar, Indonesia, unhas.ac.id; ^10^ Department of Medicine, Faculty of Medicine, Jember University, Jember, Indonesia, unej.ac.id; ^11^ Department of Medicine, Faculty of Medicine, Public Health, and Nursing, Gadjah Mada University, Yogyakarta, Indonesia, ugm.ac.id

**Keywords:** accuracy, acute ischaemic stroke, artificial intelligence, CT angiography, large vessel occlusion

## Abstract

Large vessel occlusion (LVO) requires prompt detection, and CT angiography (CTA) is frequently used due to its short acquisition time and visibility of vessels. Artificial intelligence (AI), including Viz‐LVO, CINA‐LVO, RAPID‐CTA and JLK, may be available as emerging tools for supporting timely and accurate diagnoses. This study aimed to examine and summarise the evidence of AI diagnostic performance in detecting LVO. Scopus, PubMed and ScienceDirect were utilised to search relevant articles before February 2, 2025. Studies were included in the primary outcomes analysis if they reported an overall confusion diagnostic matrix and were included in the secondary outcomes if they reported AI’s diagnostic performance by occlusion site. Of the 878 records, 11 articles were included, and 10.937 patients were identified. The pooled sensitivity and specificity were 0.87 (95% CI: 0.76–0.93) and 0.95 (95% CI: 0.91–0.97). The positive likelihood ratio (PLR) showed statistical significance (9.55 (95% CI: 5.79–13.30; *p* < 0.001; *I* ^2^: 99.9%)), whereas the negative likelihood ratio (NLR) was not significant with a pooled value of 0.14 (95% CI: 0.03–0.25; *p* < 0.624; *I* ^2^: 0%). The pooled AUC and DOR were substantial, with a pooled value of 0.87 (95% CI: 0.83–0.92; *p* < 0.001; *I* ^2^: 98.4%) and 4.69 (95% CI: 4.19–5.19; 0.001; *I* ^2^: 98.5%), respectively. Three covariates were identified (type of AI, AI software and region). However, significant heterogeneity remains in pooled PLR, AUC and DOR. The anterior circulation occlusion performed was generally acceptable, demonstrating good performance for M1 and ICA‐type *T* occlusion and moderate performance for M2 occlusion. However, poor performance was observed in ICA Type I and posterior circulation occlusion. In conclusion, AI has demonstrated excellent performance in sensitivity, specificity, PLR, AUC and DOR while showing limitations in NLR, suggesting that negative cases detected by AI require careful reevaluation through imaging review and assessment of patients’ clinical profiles to ensure better diagnostic accuracy.

## 1. Introduction

Acute ischaemic stroke (AIS) continues to have a high prevalence, morbidity and mortality worldwide [1, 2]. Most research conducted over the past decade indicates that patients presenting with AIS have a significant prevalence of LVO, accounting for more than 30% of all AIS cases [3]. For patients with an intracranial large artery occlusion in the anterior circulation, endovascular thrombectomy (EVT) has become the standard therapy up to 6 h after onset and, in some cases, up to 24 h [4, 5]. Referring to the time‐dependent effect of EVT, greater clinical outcomes depend on early diagnosis of LVO.

CT angiography (CTA) plays a crucial role in the early detection of LVO [6]. According to current clinical practice, radiologists are responsible for reading several medical images from every patient who enters their centre to determine the site of blockage in CTA. LVO must be identified as soon as possible to prevent AIS patients from experiencing a significant loss of neurological function [2]. However, there may be a longer period between a patient’s baseline imaging and the mechanical thrombectomy. Thus, a more efficient technique must be created to maximise the identification of LVO, and artificial intelligence (AI) may hold the key, as AI‐assisted interpretation may significantly reduce triage time, door‐to‐intervention notification time and door‐to‐arterial puncture time compared to conventional CTA interpretation [1, 7].

Currently, LVO detection software, including Viz LVO (Viz.ai Inc.), CINA LVO (Avicenna.AI), Rapid CTA (iSchemaView Inc.) and JLK, is available to support clinicians in making fast and accurate diagnoses [8, 9]. These tools can analyse large amounts of imaging data with high accuracy and speed by identifying patterns that neurointerventionists and radiologists may miss. This technology has demonstrated its potential to revolutionise medical imaging analysis, particularly CTA, by automating LVO detection and providing real‐time insights supporting faster clinical decision‐making [2, 8–10]. Two systematic reviews tried to evaluate the use of AI in LVO; however, no meta‐analysis has assessed the diagnostic accuracy and performance of AI in LVO [2, 8]. Additionally, the studies have not investigated the diagnostic accuracy of AI across different sites and types of LVO. Therefore, this systematic review and meta‐analysis aimed to examine and summarise the evidence of AI diagnostic performance to detect LVO.

## 2. Methods

This meta‐analysis followed the Preferred Reporting Items for Systematic Reviews and Meta‐analyses (PRISMA) reporting guidelines [11]. This study was registered on the Prospective Register of Systematic Reviews (PROSPERO) by registration number CRD42025646247.

### 2.1. Inclusion and Exclusion Criteria

We included studies that fulfilled the following requirements: (1) observational original research articles that validated the diagnostic test accuracy of AI in detecting LVO that provided a quantitative measure for diagnostic test accuracy, including true negatives (TN), false positives (FP), true positives (TP) and false negatives (FN); (2) articles compared the use of AI with ground truth comparator/gold standard; (3) articles elaborated the use of CTA. Conversely, the following articles were excluded: (1) Review‐type articles, case reports, case series, letters, commentaries and observational studies that did not observe AI in LVO were excluded from the review; (2) irretrievable full‐text; (3) published not in the English language. Studies included with no restrictions at the time of publication.

### 2.2. Search Strategy and Data Extraction

A systematic literature review was performed on Scopus, PubMed and ScienceDirect to identify relevant articles before February 2, 2025. The search terms for each database were available in Supporting Table 1. Two investigators (NMIS and SRUS) independently conducted abstract and full‐text screening using inclusion–exclusion criteria to identify eligible studies. Any conflict in the screening procedure was resolved by adding the opinion of a third party (CDT). Subsequently, the following data were extracted from eligible articles by three authors (ARS, ZRAH and CDT): (1) first author; (2) year of publication; (3) AI software; (4) type of AI; (5) location of LVO and (6) quantitative measure of diagnostic tools (TN, FP, TP and FN).

### 2.3. Quality Assessment

The tool used for assessing the risk of bias was a revised tool for the quality assessment of diagnostic accuracy studies (QUADAS‐2) [12]. The tool consisted of four domains for assessing the potential source of bias: patient selection, index test, reference standard and flow‐timing regarding the included studies. Two authors (KNC and LAO) independently evaluated the risk of bias, and any disagreements were resolved by the third party (CDT).

### 2.4. Outcomes and Findings

This study evaluates AI‐based software’s diagnostic performance with primary outcomes of quantitative analysis of pooled sensitivity, specificity, likelihood ratio, the area under the curve (AUC) and the diagnostic odds ratio (DOR) of AI in detecting LVO. The study included primary outcomes analysis when they reported the overall confusion matrix, either with an explicit figure/table or a descriptive. Secondary outcomes will incorporate AI’s diagnostic performance in the occlusion site and types‐based analysis to evaluate the use of AI in LVO based on its performance in relation to the different vascular sites of LVO. We intend to perform qualitative and quantitative measures for secondary outcomes. The studies were included in secondary outcome analysis if they reported the sensitivity and specificity regarding the specific site and/or types of occlusion, both anterior circulation and posterior circulation.

### 2.5. Statistical Analysis

Diagnostic test accuracy meta‐analysis for primary outcomes was performed using STATA/BE (v.18.0) statistical software. A random‐effects model was used to calculate sensitivity, specificity, positive likelihood ratio (PLR), negative likelihood ratio (NLR), DOR and AUC with a 95% confidence interval using ‘metan’ and ‘metadta’ packages. Cochrane’s *Q* test and *I* ^2^ statistic were used to evaluate for statistical heterogeneity between studies. If the high‐heterogeneity result was met, we intend to perform subgroup analysis to identify the potential source of heterogeneity.

For secondary outcomes, we intend to approximate the AUC and standard error of AUC from the given sensitivity and specificity provided by the author for each location of LVO with Hand’s formula [13]. We intend to perform a generic inverse variance meta‐analysis with ‘meta’ package for pooled AUC with the random‐effect model using a restricted maximum likelihood (REML) estimator and Hartung‐Knapp adjustment for accounting for the small‐study analysis [14]. The robustness of the result will be evaluated using the leave‐one‐out sensitivity technique. All analyses for secondary outcomes will be conducted in *R* Studio version 4.4.1 (PositPBC, Boston, USA).

## 3. Results

### 3.1. Literature Search and Baseline Characteristics of Included Studies

**FIGURE 1:**
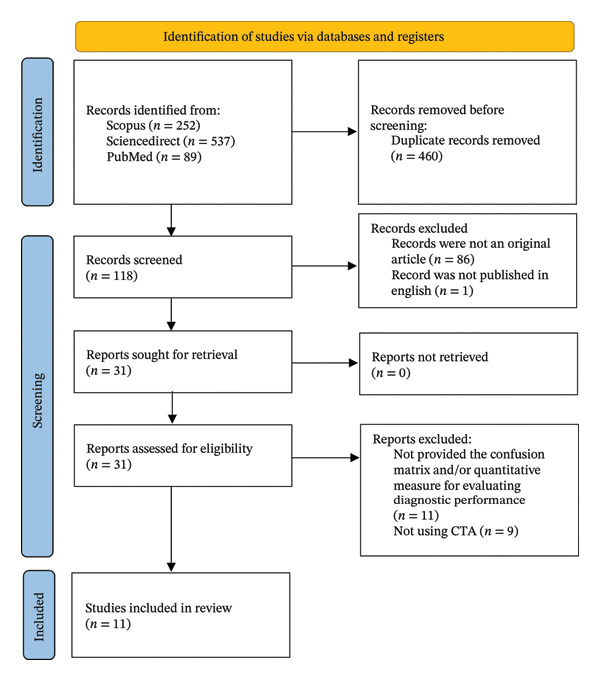
PRISMA flowchart of the selected study.

Eight hundred seventy‐eight records were identified from the initial search. After removing duplicates, investigators screened 118 records by title‐abstract. Subsequently, 31 records were sought for eligibility based on inclusion–exclusion criteria. This study included 11 articles across three databases, with the complete process available in Figure 1. Among the 11 included studies, four different AI software were evaluated. Five studies used RAPID‐LVO, three used CINA‐LVO, four used VIZ‐LVO and one incorporated JLK‐LVO. Across these studies, the ground truth for the comparator group consisted of at least two experts in neuroradiology, neurointerventional or emergency medicine. Only one study included residents as part of the comparator group. Of 11 studies, 5 elaborated on the occlusion site‐based analysis. The complete baseline characteristics of each study are available in Table 1.

**TABLE 1:** Baseline characteristics of included studies.

Author	Location and centre	Study design	Device	Type of machine	Sens (%)	Spec (%)	Ground truth comparator
Schlossman et al., 2022 [15]	USA, Single‐centre	Retrospective	RAPID‐LVO	ML	90	86	Two board‐certified neuroradiologists (9 years and 10 years of experience)
			CINA‐LVO	DL	76	98	

McLouth et al., 2021 [16]	USA, Multicentre	Retrospective	CINA‐LVO	DL	76	98	Two board‐certified neuroradiologists

Delora et al., 2024 [17]	USA, Single‐centre	Retrospective	VIZ‐LVO	DL	87	96	Neuroradiologist or neurointerventional radiologist
			RAPID‐LVO	ML	87	85	

Mellander et al., 2024 [8]	Sweden, Multicentre	Retrospective	CINA‐LVO	DL	54	93	Two board‐certified neuroradiologists or one general radiologist and one neuroradiologist

Matsoukas et al., 2023 [18]	USA, Multicentre	Prospective	VIZ‐LVO	DL	93.8	91.1	Neuroradiologist or emergency radiologist

Shalitin et al., 2020 [19]	USA, Multicentre	Retrospective	VIZ‐LVO	DL	96.3	93.7	Radiology team

Karamchandani et al., 2023 [20]	USA, Multicentre	Retrospective	VIZ‐LVO	DL	60.6	98.3	Board‐certified neuroradiologists

Dehkharghani et al., 2021 [21]	USA, Multicentre	Retrospective	RAPID‐LVO	ML	98.3	96.4	Three board‐certified neuroradiologists (11, 7 and 7 years of experience)

Amukotuwa et al., 2019 [22]	Australia, Single‐centre	Retrospective	RAPID‐LVO	ML	93.6	75.9	Neuroradiologists

Kim et al., 2024 [23]	USA, Single‐centre	Retrospective	JLK‐LVO	DL	85.8	96.9	Residents specialising in the care of ischaemic stroke from radiology, neurology, neurosurgery and emergency medicine.

Chan et al., 2023 [24]	UK, Single‐centre	Retrospective	RAPID‐LVO	ML	52.2	98.5	Two board‐certified neuroradiologists

*Note:* Sens = Sensitivity; Spec = Specificity.

Abbreviations: DL, Deep Learning; LVO, Large Vessel Occlusion; ML, Machine Learning; UK, United Kingdom; USA, United States of America.

### 3.2. Quality Assessment of Included Studies

**FIGURE 2:**
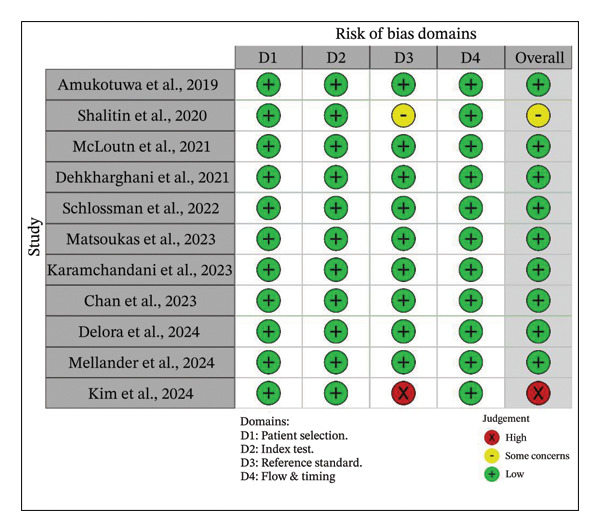
Quality assessment of the included studies based on QUADAS‐2.

Across 11 studies, Shalitin et al. were concerned about bias in reference standards (domain 3) due to not specifying the radiology team involved in the ground truth. Kim et al. were also deemed to have a high risk of bias due to their potential bias on reference standards (domain 3), as the study assessed the ground truth by only including residents. The risk of bias is depicted in Figure 2.

### 3.3. Meta‐Analysis of Diagnostic Accuracy of AI in LVO

**FIGURE 3:**
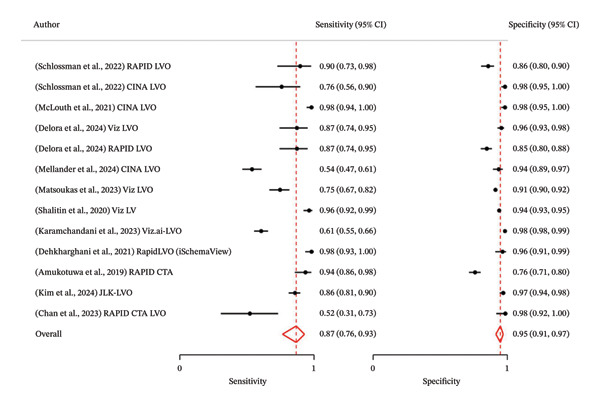
Pooled sensitivity and specificity of AI in detecting LVO.

**FIGURE 4:**
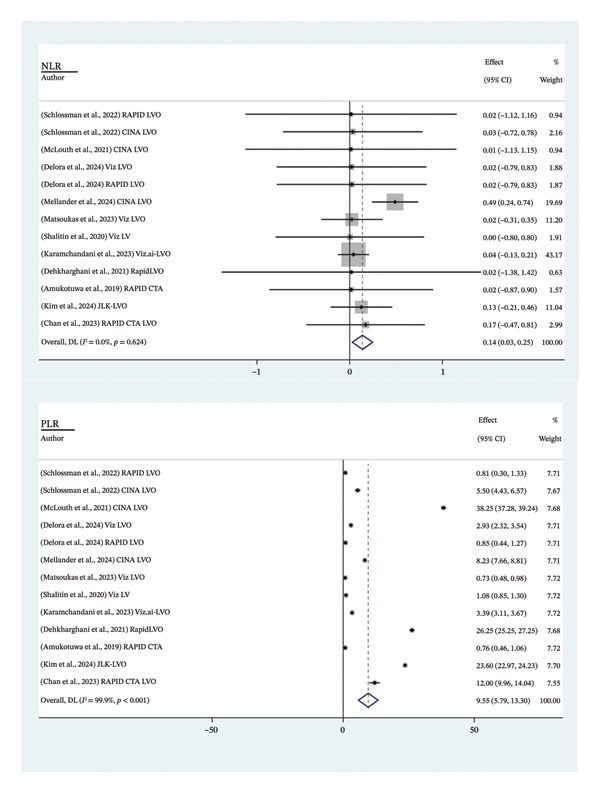
Likelihood ratio of AI in detecting LVO. NLR: negative likelihood ratio; PLR: positive likelihood ratio.

**FIGURE 5:**
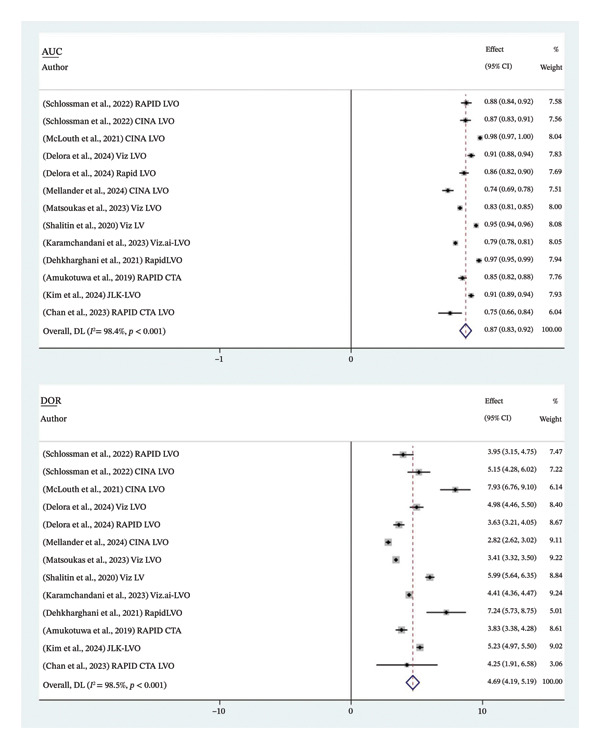
Diagnostic performance of AI in detecting LVO. DOR: diagnostic odds ratio; AUC: area under the curve.

**FIGURE 6:**
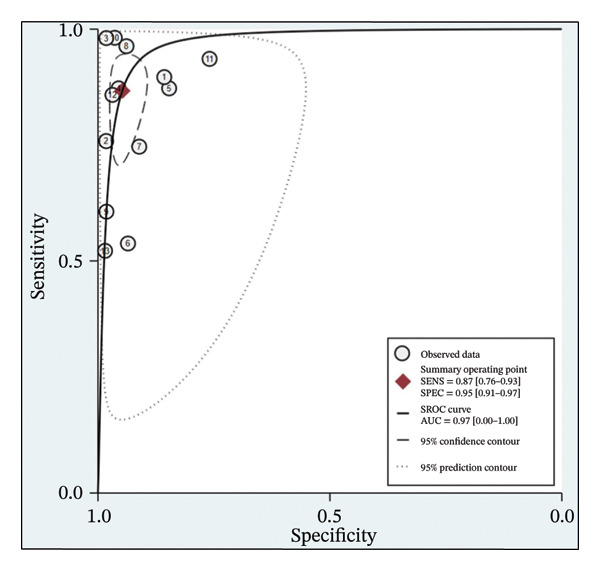
Summary receiver operating characteristic (SROC) curve.

The analysis of the 11 studies involving 13 software analyses with a total of 10.937 patients revealed the pooled sensitivity was 0.87 (95% CI: 0.76 – 0.93), while the pooled specificity was 0.95 (95% CI: 0.91–0.97) (Figure 3). The PLR showed statistical significance with a pooled value of 9.55 (95% CI: 5.79–13.30; *p* < 0.001; *I* ^2^: 99.9%), whereas the NLR was not significant with a pooled value of 0.14 (95% CI: 0.03–0.25; *p* < 0.624; *I* ^2^: 0%) (Figure 4). The pooled AUC and DOR were substantial, with a pooled value of 0.87 (95% CI: 0.83–0.92; *p* < 0.001; *I* ^2^: 98.4%) and 4.69 (95% CI: 4.19–5.19; *p* < 0.001; *I* ^2^: 98.5%), respectively (Figure 5). Overall, AI performs well as a diagnostic tool in LVO. The pooled AUC falls within the ‘good’ performance range (0.8–0.9), with the SROC curve (Figure 6) confirming that most studies cluster in the high sensitivity and specificity region, characterised by a relatively tight confidence interval, highlighting consistency across studies [23]. However, the insignificant value of NLR may reflect AI’s limitation in reliably ruling out true cases of LVO.

An exploratory analysis with subgroup analysis was conducted to identify potential sources of heterogeneity. Three covariates were identified from the covariates available within more than 10 studies (type of AI, AI software and region) (Supporting Table 2). However, despite attempts to reduce heterogeneity through subgroup analysis, significant heterogeneity remains in pooled PLR, AUC and DOR, suggesting underlying differences between studies that cannot be accounted for by the available covariates. Across different subgroups, the insignificant value of NLR was consistently observed, indicating that the predictive performance of AI in ruling out the LVO remains low regardless of the examined covariates.

### 3.4. Occlusion Site‐Based Analysis AI in LVO

Among the five included studies, all analysed AI performance in detecting occlusions in the anterior circulation, while only one study examined the posterior circulation. On the posterior circulation, a retrospective study by Mellander et al. revealed an unacceptable value of the diagnostic performance of AI. The performance of AI in posterior LVO yielded a sensitivity of 0.0 (95% CI: 0.0–0.2) and a specificity of 1.00 (95% CI: 0.99–1.00). Further specified sites of the basilar artery and P1 segment of the posterior cerebral artery yielded an unchanged result, further highlighting the poor performance in this area [8].

**FIGURE 7:**
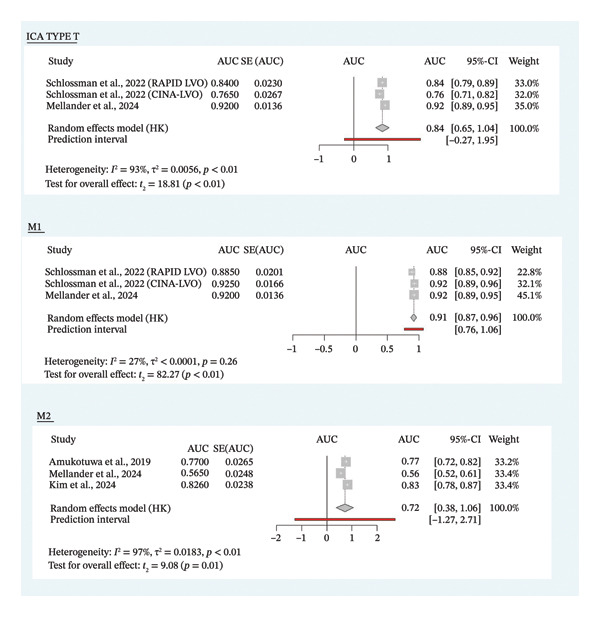
Pooled area under the curve (AUC) of AI’s performance on different anatomical sites. ICA‐T = internal carotid artery terminus; M1 = first segment of the middle cerebral artery; M2 = second segment of the middle cerebral artery.

For the anterior circulation, four studies with five analyses elaborated on the good sensitivity performance of ICA type *T* occlusion, with a range of 0.84–1.00 [8, 15, 18, 22]. The pooled AUC from three analyses, which provided the sensitivity and specificity, reveals good diagnostic performance with a value of 0.84 (95% CI: 0.65 to 1.04; *p* < 0.01; Figure 7 [8, 15]. However, this result was not robust based on the leave‐one‐out analysis, as excluding Schlossman et al. (RAPID‐LVO) rendered the result nonsignificant (Supporting Figure 1). Although the performance for ICA type *T* occlusion was excellent, Mellander et al. reported an unacceptably low sensitivity for detecting ICA type I occlusion, highlighting substantial challenges in identifying this specific occlusion subtype [8].

On the diagnostic performance of middle cerebral artery (MCA) segment M1, the overall sensitivity for M1 was high, ranging from 0.87 to 0.93 [8, 15, 18, 22]. The pooled AUC from three studies was 0.913 (95% CI: 0.87 to 0.96; *p* < 0.01; Figure 7) with overall robustness based on leave‐one‐out analysis (Supporting Figure 2) [15, 18]. For the M2 segment of MCA, the results across four studies with five analyses yielded varying results. The sensitivity ranged from 0.3 to 0.86, with specificity ranging from 0.68 to 1.00 [8, 15, 22, 25]. Pooled AUC from three studies revealed a significant result with a value of 0.72 (95% CI: 0.38 to 1.06; *p* = 0.01; Figure 7) [8, 22, 25]. Leave‐one‐out analysis confirmed that the overall significance value was not robust, as excluding Amukotuwa et al. and Kim et al. resulted in a shifting of the significant value (Supporting Figure 3).

Overall, while AI demonstrated a good diagnostic performance in detecting LVO, there were varying results across different sites and types of occlusions (Table 2). The anterior circulation occlusion performed was generally acceptable, demonstrating good performance for M1 and ICA type *T* occlusions, and moderate performance for M2 occlusions. However, there was poor performance on ICA type I and posterior circulation occlusion, highlighting the need for further optimisation and validation to improve AI performance.

**TABLE 2:** AI’s performance in occlusion site‐based analysis.

Author (year)	Sensitivity	Specificity
*ICA type T*		
Amukotuwa et al., 2019 (RAPID‐LVO)	0.969	—
Schlossman et al., 2022 (RAPID‐LVO)	0.82	0.86
Schlossman et al., 2022 (CINA LVO)	0.55	0.98
Matsoukas et al., 2023 (Viz‐LVO)	1	—
Mellander et al., 2024 (CINA‐LVO)	0.84	1

*M1*		
Amukotuwa et al., 2019 (RAPID‐LVO)	0.913	—
Schlossman et al., 2022 (RAPID‐LVO)	0.91	0.86
Schlossman et al., 2022 (CINA LVO)	0.87	0.98
Matsoukas et al., 2023 (Viz‐LVO)	0.87	0.97
Mellander et al., 2024 (CINA‐LVO)	0.87	—

*M2*		
Amukotuwa et al., 2019 (RAPID LVO)	0.86	0.68
Schlossman et al., 2022 (RAPID LVO)	0.8	—
Schlossman et al., 2022 (CINA LVO)	0.3	—
Mellander et al., 2024 (CINA‐LVO)	0.13	1
Kim et al., 2024 (JLK‐LVO)	0.692	0.96

*Note:* M1 = first segment of the middle cerebral artery; M2 = second segment of the middle cerebral artery.

Abbreviation: ICA‐T, internal carotid artery terminus.

## 4. Discussion

LVO represents a high‐mortality condition that demands a swift and accurate diagnostic modality to improve patient outcomes. AI has emerged as a novel diagnostic technology advancement for identifying LVO stroke, with a quantitative review of its accuracy remaining uncharted [2, 23]. Ultimately, this study sought to provide a qualitative and quantitative evaluation of AI in LVO.

In our study, we found that all of the AI software subsets generally have good diagnostic performance. This advancement is primarily driven by the natural ability of AI to identify subtle patterns and anomalies in CTA rapidly [26]. Referring to ‘time is brain’, the immediate detection by AI may accelerate door‐to‐treatment times, which significantly improves radiology CTA report turnaround times [27]. This acceleration is particularly beneficial for healthcare in daily practice for significantly synchronising stroke triage, as current guidelines suggest rapid diagnosis within 6 h of symptom onset for acute anterior LVO and up to 24 h for selected cases with favourable imaging profiles [28]. In addition, this advancement may also help bridge the learning curve for new operators. In situations where operators are unavailable or their expertise is limited, AI can provide critical support, ensuring rapid and accurate diagnostic assistance in such urgent scenarios.

Despite its advancement, our study highlights the nonsignificant result of NLR in overall performance. Across all covariates of the subgroup, the result showed consistent reports. This limitation suggests that while AI can accurately identify positive cases, its ability to rule out negative cases precisely may be less pronounced. Therefore, we suggest several recommendations. First, there is a need for additional review by a radiologist or neurologist in cases where ML detects a negative LVO, alongside clinical re‐evaluation through additional neurological assessments [14, 29]. Second, we encourage follow‐up imaging for oligosymptomatic LVO and, if necessary, a multidisciplinary team review to provide comprehensive judgement in ambiguous cases, ensuring no LVO is left behind [19, 29]. Third, continuous software updates and extensive research on AI are crucial to improving diagnostic accuracy, aiming to provide significant results for the NLR [26].

Our study also highlights the varying results across different types and sites of LVO. Notably, AI has shown poor performance in the posterior circulation. This result may be a consequence of AI algorithms that were not specifically designed or trained to detect these occlusions [8]. Although posterior circulation LVO is well recognised as being less common than anterior LVO, with an estimated incidence ratio of approximately 1:5, it remains an inherently challenging case to identify, even for experienced neuroradiologists and neurointerventionalists. Given their significant clinical impact, AI tools that improve detection in these cases would be highly beneficial [30, 31]. Thus, future advancements should prioritise enhancing AI performance in the posterior circulation.

However, even within anterior LVO, our findings reveal a significant variability, likely influenced by the specific AI software used. For M1 occlusion, all studies across different software platforms consistently demonstrated strong performance. In contrast, for ICA‐T occlusions, excluding Schlossman et al., which utilised RAPID, resulted in a loss of statistical significance, suggesting that RAPID may outperform CINA, as the two other analyses (Schlossman et al. and Mellander et al.) employed CINA. Additionally, ICA type‐I occlusion, involving the distal ICA beyond the ophthalmic artery but sparing the terminus, exhibited an unacceptable diagnostic value, with no cases detected by AI. This poor performance may be derived from insufficient representation of ICA type‐I occlusion in the algorithm’s training data, despite claims that the software is designed for distal ICA occlusion detection [8]. The greatest variability, however, was observed in M2 occlusion, where sensitivity and specificity fluctuated significantly across different AI models. For instance, Mellander et al. (using CINA‐LVO) reported very low sensitivity but high specificity, while Kim et al. (using JLK‐LVO) demonstrated moderate sensitivity with high specificity. These discrepancies underscore the substantial impact of software differences on overall diagnostic performance, reinforcing the need for standardised validation across various AI platforms to ensure consistent and reliable detection of LVO.

To the best of our knowledge, this is the first diagnostic meta‐analysis of AI in LVO. We also provide insights into specific sites and types of LVO, offering a more in‐depth analysis to strengthen the findings. However, several limitations of this study need to be acknowledged. Our study is limited to the availability of individual‐level data in sites and type analysis provided by the included literature. We only approximate the overall diagnostic performance by qualitative findings and quantitative AUC using Hand’s estimator. Our study also only explores the subgroup effect of the diagnostic performance by limited covariates without any relief in heterogeneity found, considering the limited covariates provided by the literature within more than 10 studies. Future direction should address discrepancies across AI software while balancing sensitivity and specificity for each specific site and LVO types in tool development. Standardisation is essential, as prioritising one metric over the other could lead to alert fatigue or risk missing LVO cases. Additionally, implementing relative vessel density thresholds may offer a potential solution for improving detection accuracy.

## 5. Conclusion

AI has emerged as a promising diagnostic tool in supporting the detection of LVO. AI has demonstrated excellent performance in sensitivity, specificity, PLR, AUC and DOR while showing limitations in NLR. These limitations suggest that negative cases detected by AI require careful re‐evaluation through imaging review and assessment of patients’ clinical profiles to ensure better diagnostic accuracy. Together, these advancements mark a pivotal shift in technology’s involvement in revolutionising healthcare.

## Funding

This study received no external funding.

## Ethics Statement

The authors have nothing to report.

## Conflicts of Interest

The authors declare no conflicts of interest.

## Supporting Information

The Supporting Information provides additional methodological and analytical details supporting this study. Supporting Table S1 presents the detailed search strategies used across multiple databases. Supporting Table S2 summarises the results of subgroup analyses. Supporting Figures 1–3 represent the leave‐one‐out sensitivity analyses for different occlusion types.

## Supporting information


**Supporting Information** Additional supporting information can be found online in the Supporting Information section.

## Data Availability

All data generated or analyzed during this study are included in this published article and its supporting information. Additional information can be made available from the corresponding author upon reasonable request.
