# Supplementary material for: Diagnostic Test Accuracy of Artificial Intelligence in Large Vessel Occlusion: A Systematic Review and Meta‐Analysis
Source: Neurol Res Int. 2026 Apr 21;2026:5709868. doi: 10.1155/nri/5709868 (PMC13100493; doi:10.1155/nri/5709868)
Supplement: Supplementary file 1 — Supporting Information Additional supporting information can be found online in the Supporting Information section. [file NRI-2026-5709868-s001.docx]

**Supplementary Table 1.** Search strategy

| **Database** | **Search fields & full search string** |
| --- | --- |
| **PubMed** | **Search field:** All fields with MeSH and free-text terms (("Stroke"[Mesh] OR stroke*[tiab] OR "ischemic stroke"[tiab] OR "ischaemic stroke"[tiab]) AND ("computed tomography angiography"[tiab] OR "CT angiograph*"[tiab] OR CTA[tiab]) AND ("large vessel occlusion"[tiab] OR "large-vessel occlusion"[tiab] OR LVO[tiab]) AND ("Artificial Intelligence"[Mesh] OR "artificial intelligence"[tiab] OR "deep learning"[tiab] OR "machine learning"[tiab] OR "neural network*"[tiab] OR "computer-aided detection"[tiab] OR "computer-assisted"[tiab])) |
| **Scopus** | **Search field:** TITLE-ABS-KEY TITLE-ABS-KEY ((stroke OR "ischemic stroke" OR "ischaemic stroke") AND ("computed tomography angiography" OR "CT angiograph*" OR CTA) AND ("large vessel occlusion" OR "large-vessel occlusion" OR LVO) AND ("artificial intelligence" OR "deep learning" OR "machine learning" OR "neural network*" OR "computer-aided detection" OR "computer-assisted")) |
| **ScienceDirect (Elsevier)** | **Search field:** Title, abstract, and keywords ("stroke" OR "ischemic stroke" OR "ischaemic stroke") AND ("computed tomography angiography" OR "CT angiography" OR CTA) AND ("large vessel occlusion" OR "large-vessel occlusion" OR LVO) AND ("artificial intelligence" OR "deep learning" OR "machine learning" OR "neural network" OR "computer-aided detection" OR "computer-assisted") |

**Supplementary Table 2.** Subgroup analysis result

|  | **Number of studies** | **Sensitivity**  **(95% CI)** | **Specificity (95% CI)** | **NLR**  **(95% CI;**  **p-value; I^2^)** | **PLR**  **(95% CI;**  **p-value; I^2^)** | **AUC**  **(95% CI;**  **p-value; I^2^)** | **logDOR**  **(95% CI;**  **p-value; I^2^)** |
| --- | --- | --- | --- | --- | --- | --- | --- |
| **AI-type** |  |  |  |  |  |  |  |
| RAPID-LVO | 5 | 0.9  (0.74-0.96) | 0.91  (0.79-0.96) | 0.08  (-0.32-0.47;  p = 0.998;  I^2^ = 0%) | 8.10  (2.12 – 14.08; p < 0.001;  I^2^ = 99.8%) | 0.87  (0.8 – 0.93;  p < 0.001;  I^2^ = 94.1%) | 4.32  (3.54 – 5.10;  p < 0.001;  I^2^ = 80.6%) |
| CINA-LVO | 3 | 0.85  (0.5 – 0.97) | 0.97  (0.94 – 0.99) | 0.43  (0.19 – 0.66;  p = 0.406;  I^2^ = 0%) | 17.33  (-1.7 – 36.35); p < 0.001;  I^2^ = 99.9%) | 0.86  (0.72 – 1.01;  p < 0.001;  I^2^ = 98.4) | 5.26  (2.39 – 8.12;  p < 0.001;  I^2^ = 97.9%) |
| VIZ -LVO | 4 | 0.84  (0.64 – 0.94) | 0.95  (0.92 – 0.97) | 0.04  (-0.11 – 0.18;  p = 0.999;  I^2^ = 0%) | 2.02  (0.7 – 3.34);  p < 0.001;  I^2^ = 98.8%) | 0.87  (0.78 – 0.96;  p < 0.001;  I^2^ = 99.4%) | 4.68  (3.9 – 5.45;  p < 0.001;  I^2^ = 99.4%) |
| JLK-LVO | 1 | 0.86 | 0.97 | 0.12 | 23.6 | 0.91 | 5.23 |
| **Type of AI software** |  |  |  |  |  |  |  |
| DL | 8 | 0.84  (0.7 – 0.93) | 0.96  (0.94 – 0.98) | 0.15  (-0.01 – 0.31; p = 0.209;  I^2^ = 27.6% | 10.45  (5.2 – 15.7);  p < 0.001;  I^2^ = 99.9%) | 0.87  (0.82 – 0.92;  p < 0.001;  I^2^ = 98.9%) | 4.85  (4.23 – 5.47;  p < 0.001;  I^2^ = 99.1%) |
| ML | 5 | 0.9  (0.74-0.96) | 0.91  (0.79-0.96) | 0.08  (-0.32-0.47;  p = 0.998;  I^2^ = 0%) | 8.10  (2.12 – 14.08; p < 0.001;  I^2^ = 99.8%) | 0.87  (0.8 – 0.93;  p < 0.001;  I^2^ = 94.1%) | 4.32  (3.54 – 5.10;  p < 0.001;  I^2^ = 80.6%) |
| **Region** |  |  |  |  |  |  |  |
| USA | 10 | 0.89  (0.81 – 0.95) | 0.95  (0.92 – 0.97) | 0.05  (-0.08-0.18;  p = 1.0;  I^2^ = 0%) | 10.32  (5.61 – 15.04; p < 0.001;  I^2^ = 99.9%) | 0.9  (0.85 – 0.94;  p < 0.001;  I^2^ = 98.6%) | 5.00  (4.47 – 5.54;  p < 0.001;  I^2^ = 98.5%) |
| Non-USA | 3 | 0.71 (0.37-0.91) | 0.91 (0.78-0.97) | 0.42 (0.19=0.64; p = 0.43; I^2^ = 0%) | 6.94 (0.71-13.17; p <0.001; I^2^ = 99.7%) | 0.78 (0.70 – 0.87;  p < 0.001;  I^2^ = 88.7%) | 3.41 (2.50 – 4.33;  p < 0.001;  I^2^ = 88.5%) |


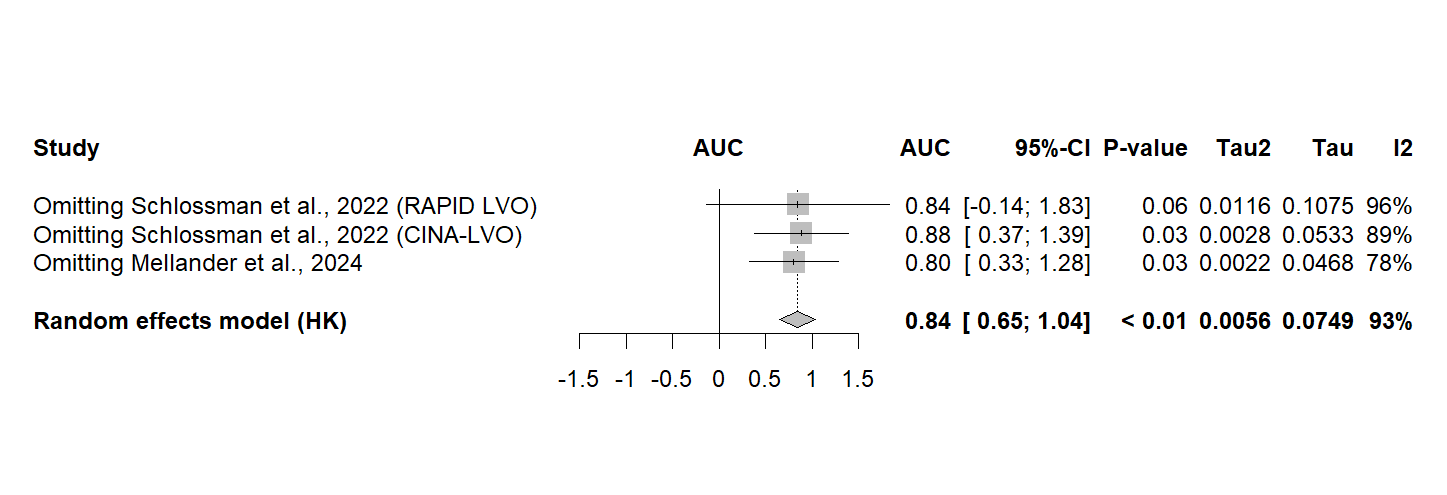


**Supplementary Figure 1.** Leave-one-out of ICA type T analysis


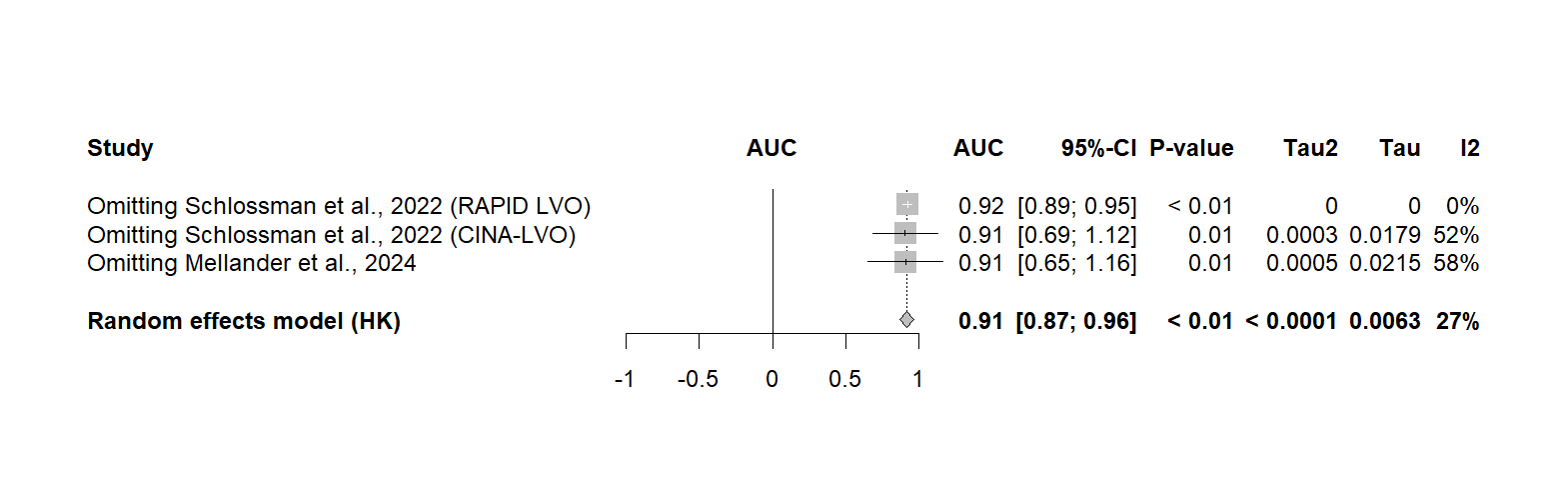


**Supplementary Figure 2.** Leave-one-out of M1 analysis


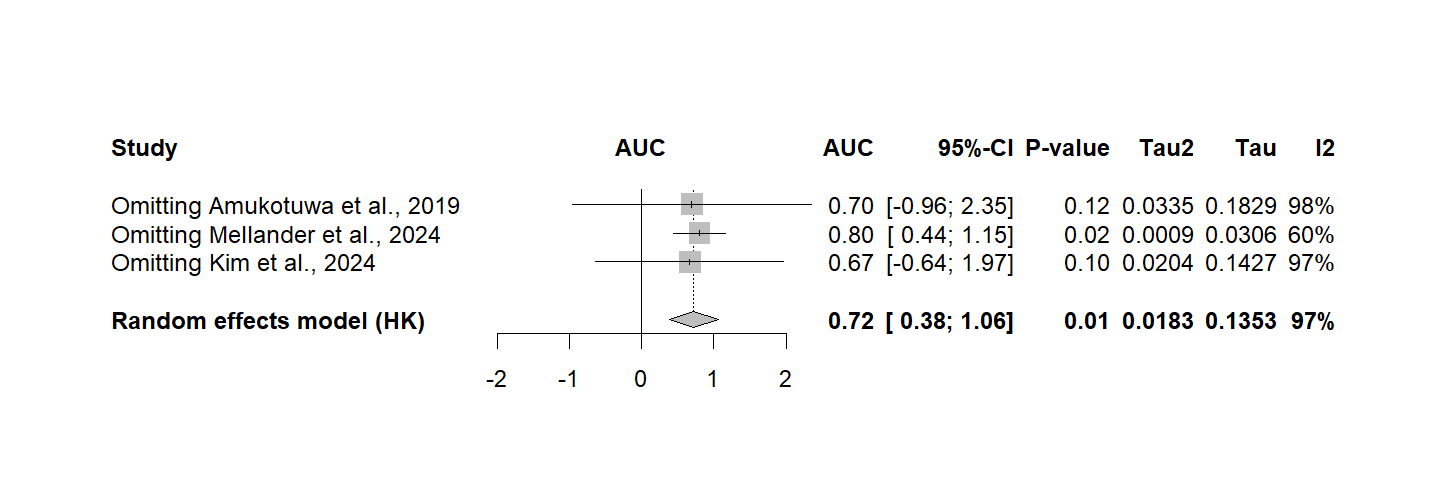


**Supplementary Figure 3.** Leave-one-out of M2 analysis
